# The genome sequence of the bulrush Neoascia, *Neoascia interrupta *(Meigen, 1822)

**DOI:** 10.12688/wellcomeopenres.20353.1

**Published:** 2023-11-23

**Authors:** Steven Falk, Katie J. Woodcock, University of Oxford and Wytham Woods Genome Acquisition Lab, Darwin Tree of Life Barcoding collective, Wellcome Sanger Institute Tree of Life programme, Wellcome Sanger Institute Scientific Operations: DNA Pipelines collective, Tree of Life Core Informatics collective, Darwin Tree of Life Consortium

**Affiliations:** 1 Independent researcher, Kenilworth, England, UK; 2 Tree of Life, Wellcome Sanger Institute, Hinxton, England, UK

**Keywords:** Neoascia interrupta, bulrush Neoascia, genome sequence, chromosomal, Diptera

## Abstract

We present a genome assembly from an individual female *Neoascia interrupta* (the bulrush Neoascia; Arthropoda; Insecta; Diptera; Syrphidae). The genome sequence is 601.9 megabases in span. Most of the assembly is scaffolded into 4 chromosomal pseudomolecules, including the X sex chromosome. The mitochondrial genome has also been assembled and is 17.76 kilobases in length. Gene annotation of this assembly on Ensembl identified 22,086 protein coding genes.

## Species taxonomy

Eukaryota; Metazoa; Eumetazoa; Bilateria; Protostomia; Ecdysozoa; Panarthropoda; Arthropoda; Mandibulata; Pancrustacea; Hexapoda; Insecta; Dicondylia; Pterygota; Neoptera; Endopterygota; Diptera; Brachycera; Muscomorpha; Eremoneura; Cyclorrhapha; Aschiza; Syrphoidea; Syrphidae; Eristalinae; Milesiini; *Neoascia;* Neoascia interrupta (Meigen, 1822)(NCBI:txid2867103).

## Background

The bulrush *Neoascia, Neoascia interrupta* (Meigen, 1822), is a predominantly black and notably small species of hoverfly found in Northern and Central Europe and Western Siberia ( van Veen, 2014). In the UK *N. interrupta* are found between the months of April and September and peak in numbers during June ( Stubbs & Falk, 2002). After a relatively recent addition to the UK species list in 1981, records of *N. interrupta* are most frequent in the East of England though individuals have been documented across Southern England, Wales and into South Yorkshire ( Ball & Morris, 2000; Ball & Morris, 2015). Adults display a preference for vegetation surrounding ponds, marshes, ditches and canals which are often rich in bulrush reeds, hence their common name, the bulrush *Neoascia* ( Stubbs & Falk, 2002). The species has additionally been noted occupying semi-brackish ditches in coastal areas of Kent and Essex ( Stubbs & Falk, 2002). *N. interrupta* is distinguished from other *Neoascia* hoverflies by the presence of yellow spots on the edges of abdominal tergite 4, which are lacking in other members of the genus ( Stubbs & Falk, 2002; van Veen, 2014). Females have a club-shaped lower abdomen, contrastingly males are more streamlined in appearance, though both sexes are noted for their distinct wasp-like narrow waists ( Ball & Morris, 2015). Species specific records of breeding and larval development are limited, though larvae of the *Neoascia* genus have been found in wet manure, compost and decaying vegetation associated with the banks of ponds and ditches ( Ball & Morris, 2015; Rotheray, 1993). Adult *N. interrupta* are frequent flower visitors and can be spotted flying slowly among low-growing flowers such as forget-me-nots and fool’s watercress ( Stubbs & Falk, 2002; van Veen, 2014). The completed genome sequence for *Neoascia interrupta* as part of the Darwin Tree of Life Project provides the opportunity to further investigate this understudied hoverfly species.

## Genome sequence report

**Figure 1.**
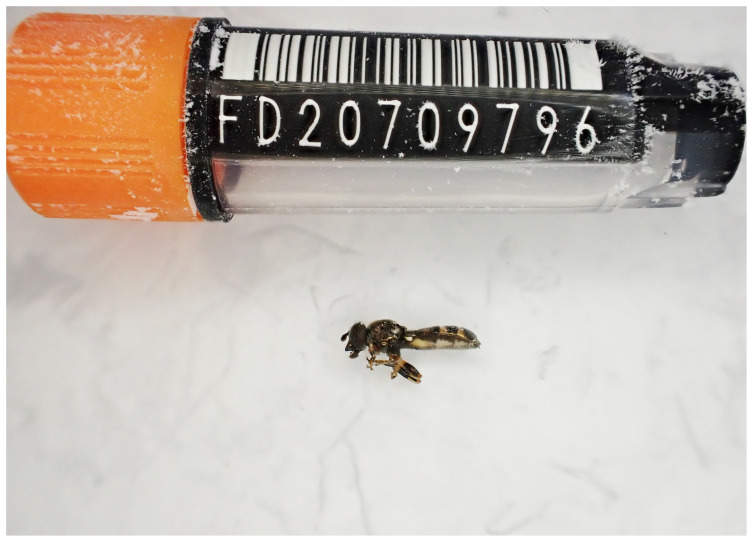
Photograph of the *Neoascia interrupta* (idNeoInte1) specimen used for genome sequencing.

The genome was sequenced from one female *Neoascia interrupta* ( Figure 1) collected from Wytham Woods, Oxfordshire, UK (51.76, –1.34). A total of 45-fold coverage in Pacific Biosciences single-molecule HiFi long reads was generated. Primary assembly contigs were scaffolded with chromosome conformation Hi-C data. Manual assembly curation corrected 134 missing joins or mis-joins and removed one haplotypic duplication, reducing the scaffold number by 60.61%, and increasing the scaffold N50 by 14.82%.

**Figure 2.**
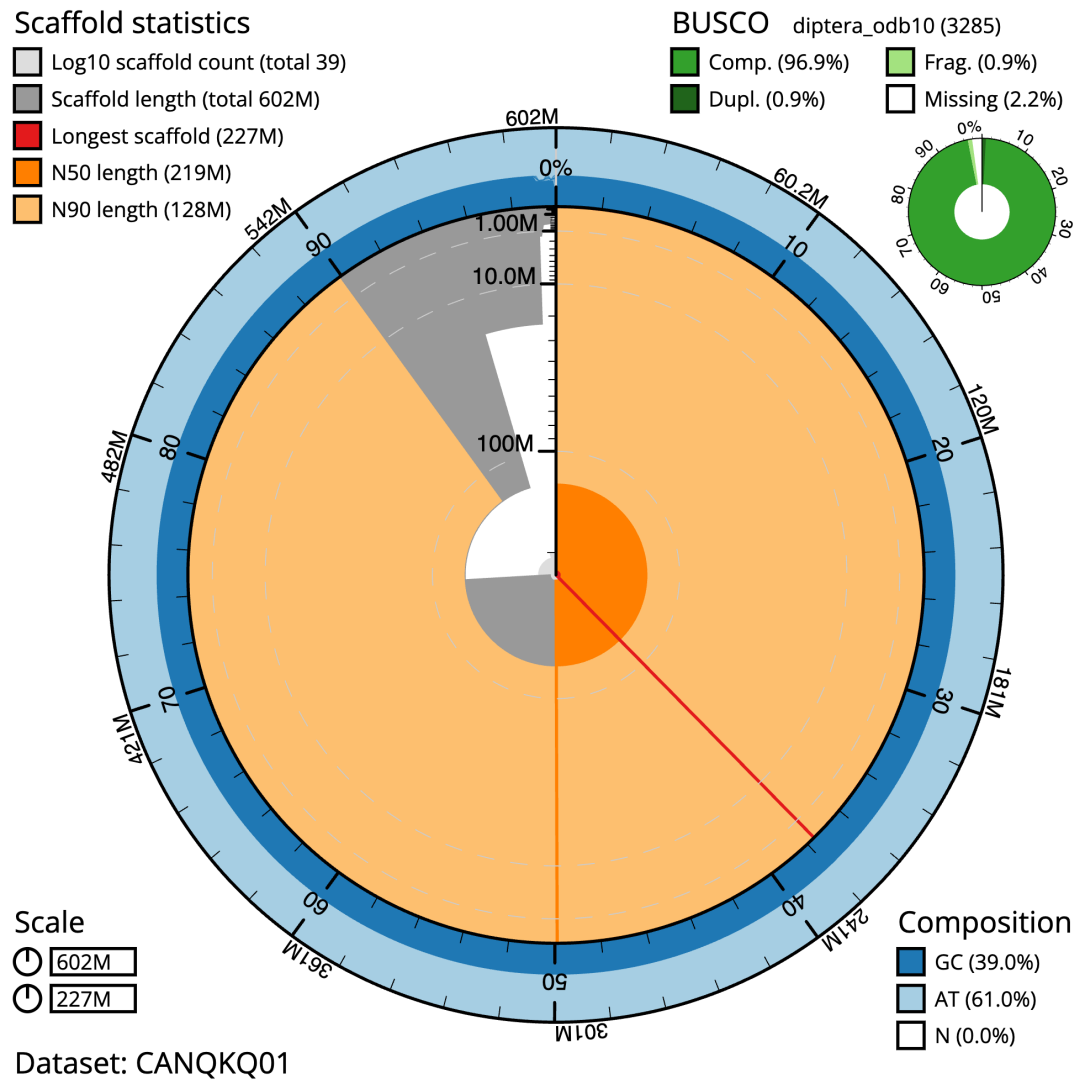
Genome assembly of *Neoascia interrupta*, idNeoInte1.1: metrics. The BlobToolKit Snailplot shows N50 metrics and BUSCO gene completeness. The main plot is divided into 1,000 size-ordered bins around the circumference with each bin representing 0.1% of the 601,923,271 bp assembly. The distribution of scaffold lengths is shown in dark grey with the plot radius scaled to the longest scaffold present in the assembly (226,676,804 bp, shown in red). Orange and pale-orange arcs show the N50 and N90 scaffold lengths (219,059,534 and 128,085,943 bp), respectively. The pale grey spiral shows the cumulative scaffold count on a log scale with white scale lines showing successive orders of magnitude. The blue and pale-blue area around the outside of the plot shows the distribution of GC, AT and N percentages in the same bins as the inner plot. A summary of complete, fragmented, duplicated and missing BUSCO genes in the diptera_odb10 set is shown in the top right. An interactive version of this figure is available at https://blobtoolkit.genomehubs.org/view/Neoascia%20interrupta/dataset/CANQKQ01/snail.

**Figure 3.**
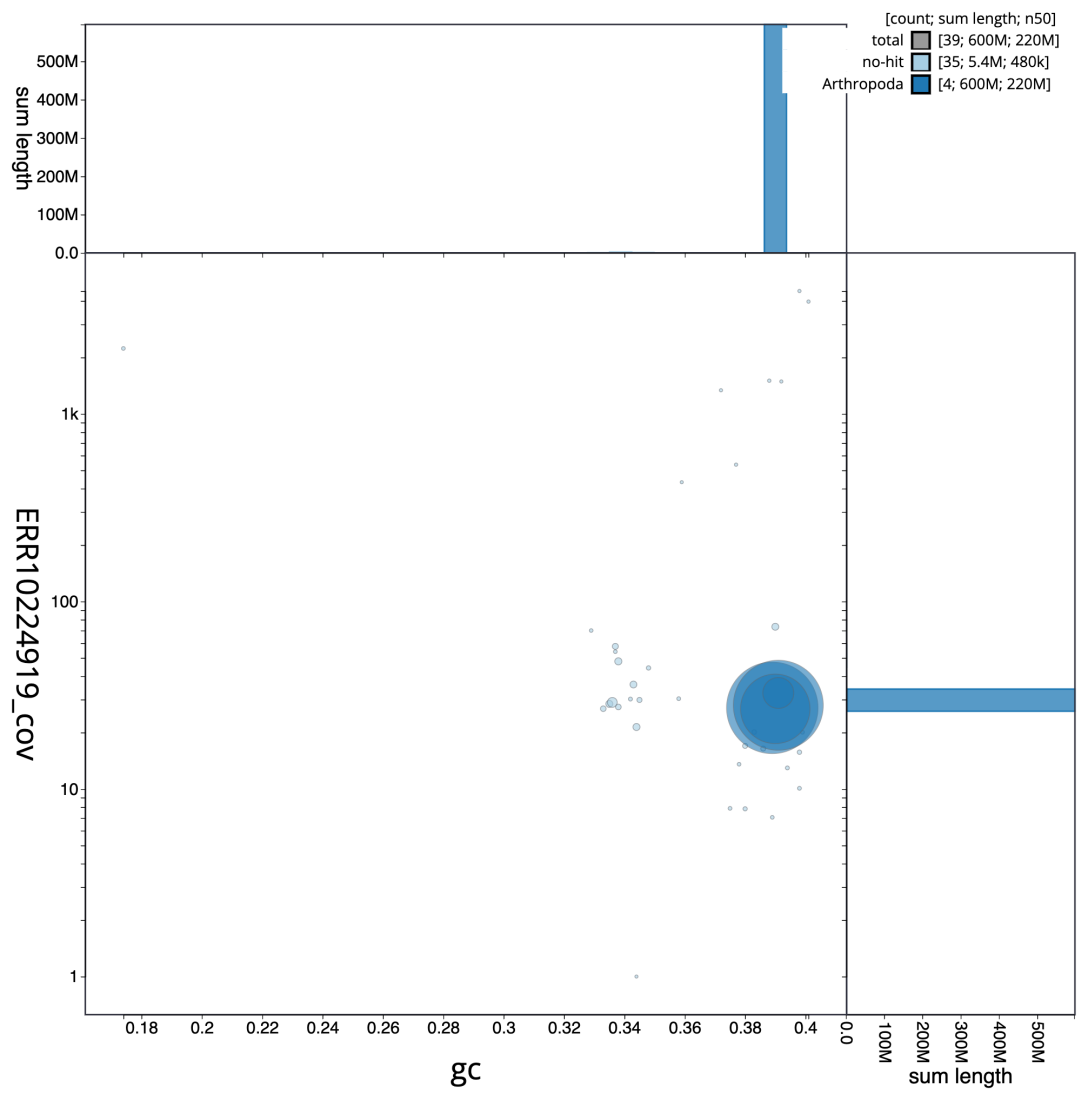
Genome assembly of *Neoascia interrupta*, idNeoInte1.1: BlobToolKit GC-coverage plot. Scaffolds are coloured by phylum. Circles are sized in proportion to scaffold length. Histograms show the distribution of scaffold length sum along each axis. An interactive version of this figure is available at https://blobtoolkit.genomehubs.org/view/Neoascia%20interrupta/dataset/CANQKQ01/blob.

**Figure 4.**
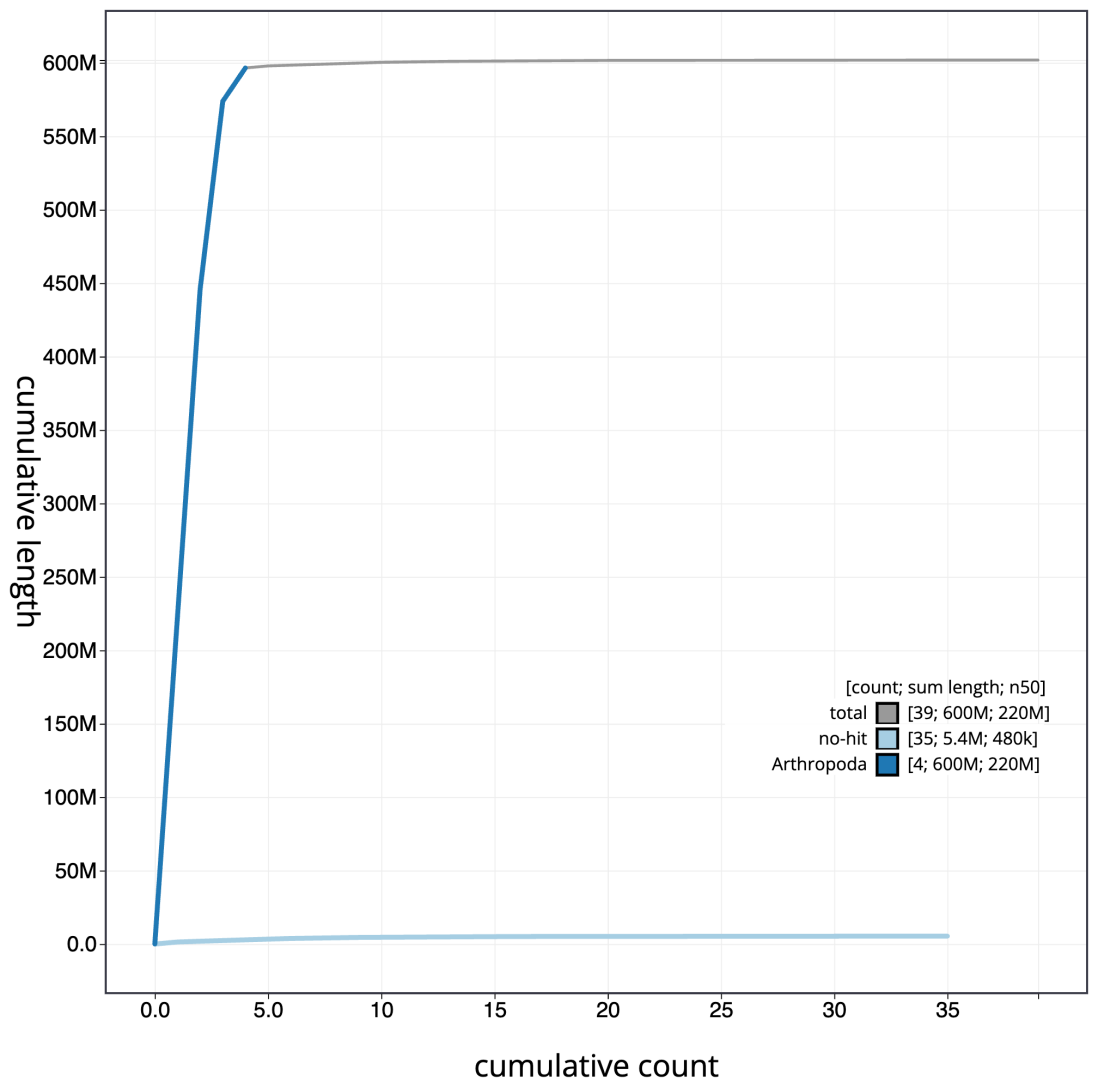
Genome assembly of *Neoascia interrupta*, idNeoInte1.1: BlobToolKit cumulative sequence plot. The grey line shows cumulative length for all scaffolds. Coloured lines show cumulative lengths of scaffolds assigned to each phylum using the buscogenes taxrule. An interactive version of this figure is available at https://blobtoolkit.genomehubs.org/view/Neoascia%20interrupta/dataset/CANQKQ01/cumulative.

**Figure 5.**
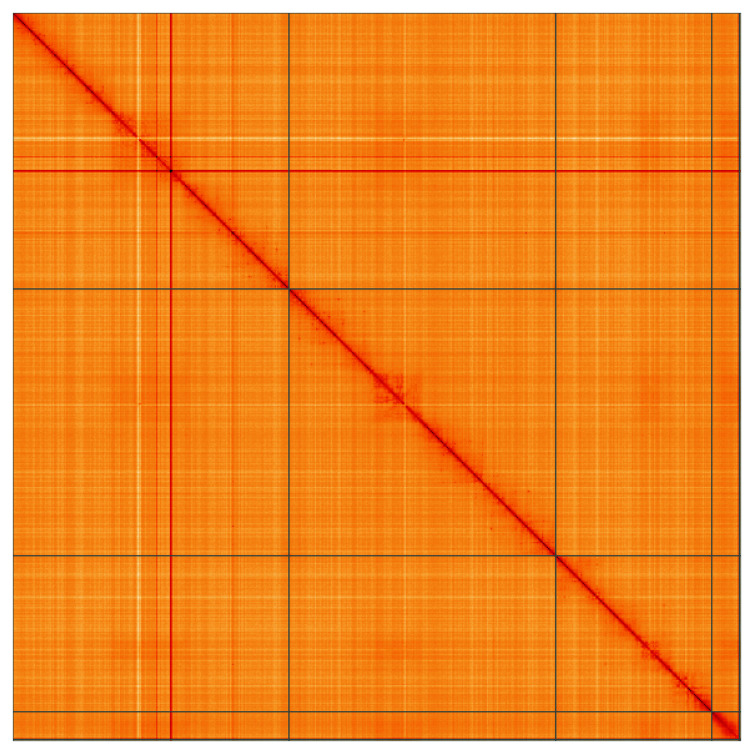
Genome assembly of *Neoascia interrupta*, idNeoInte1.1: Hi-C contact map of the idNeoInte1.1 assembly, visualised using HiGlass. Chromosomes are shown in order of size from left to right and top to bottom. An interactive version of this figure may be viewed at https://genome-note-higlass.tol.sanger.ac.uk/l/?d=LMw6NIp_REaXbeYMTvjmaw.

The final assembly has a total length of 601.9 Mb in 38 sequence scaffolds with a scaffold N50 of 219.1 Mb ( Table 1). The snailplot in Figure 2 provides a summary of the assembly statistics, while the distribution of assembly scaffolds on GC proportion and coverage is shown in Figure 3. The cumulative assembly plot in Figure 4 shows curves for subsets of scaffolds assigned to different phyla. Most (99.17%) of the assembly sequence was assigned to 4 chromosomal-level scaffolds, representing 3 autosomes and the X sex chromosome. Chromosome-scale scaffolds confirmed by the Hi-C data are named in order of size ( Figure 5; Table 2). While not fully phased, the assembly deposited is of one haplotype. Contigs corresponding to the second haplotype have also been deposited. The mitochondrial genome was also assembled and can be found as a contig within the multifasta file of the genome submission.

**Table 1.** Genome data for *Neoascia interrupta*, idNeoInte1.1.

Project accession data		
Assembly identifier	idNeoInte1.1	
Species	*Neoascia interrupta*	
Specimen	idNeoInte1	
NCBI taxonomy ID	2867103	
BioProject	PRJEB55984	
BioSample ID	SAMEA10166789	
Isolate information	idNeoInte1, whole organism (DNA sequencing and Hi-C data)	
Assembly metrics *		*Benchmark*
Consensus quality (QV)	63.2	*≥ 50*
*k*-mer completeness	100%	*≥ 95%*
BUSCO **	C:96.9%[S:96.0%,D:0.9%], F:0.9%,M:2.2%,n:3,285	*C ≥ 95%*
Percentage of assembly mapped to chromosomes	99.17%	*≥ 95%*
Sex chromosomes	X chromosome	*localised homologous pairs*
Organelles	Mitochondrial genome assembled	*complete single alleles*
Raw data accessions		
PacificBiosciences SEQUEL II	ERR10224919	
Hi-C Illumina	ERR10297812	
Genome assembly		
Assembly accession	GCA_947623515.1	
*Accession of alternate haplotype*	GCA_947622765.1	
Span (Mb)	601.9	
Number of contigs	373	
Contig N50 length (Mb)	4.0	
Number of scaffolds	38	
Scaffold N50 length (Mb)	219.1	
Longest scaffold (Mb)	226.7	
Genome annotation		
Number of protein-coding genes	22,086	
Number of gene transcripts	22,574	

* Assembly metric benchmarks are adapted from column VGP-2020 of “Table 1: Proposed standards and metrics for defining genome assembly quality” from ( Rhie *et al.*, 2021). ** BUSCO scores based on the diptera_odb10 BUSCO set using v5.3.2. C = complete [S = single copy, D = duplicated], F = fragmented, M = missing, n = number of orthologues in comparison. A full set of BUSCO scores is available at https://blobtoolkit.genomehubs.org/view/Neoascia%20interrupta/dataset/CANQKQ01/busco.

**Table 2.** Chromosomal pseudomolecules in the genome assembly of *Neoascia interrupta*, idNeoInte1.

INSDC accession	Chromosome	Length (Mb)	GC%
OX392462.1	1	226.68	39.0
OX392463.1	2	219.06	39.0
OX392464.1	3	128.09	39.0
OX392465.1	X	22.7	39.0
OX392466.1	MT	0.02	17.5

The estimated Quality Value (QV) of the final assembly is 63.2 with *k*-mer completeness of 100%, and the assembly has a BUSCO v5.3.2 completeness of 96.9% (single = 96.0%, duplicated = 0.9%), using the diptera_odb10 reference set ( *n* = 3,285).

Metadata for specimens, barcode results, spectra estimates, sequencing runs, contaminants and pre-curation assembly statistics are given at https://links.tol.sanger.ac.uk/species/2867103.

## Genome annotation report

The *Neoascia interrupta* genome assembly (GCA_947623515.1) was annotated using the Ensembl rapid annotation pipeline ( Table 1; https://rapid.ensembl.org/Neoascia_interrupta_GCA_947623515.1/Info/Index). The resulting annotation includes 22,574 transcribed mRNAs from 22,086 protein-coding genes.

## Methods

### Sample acquisition and nucleic acid extraction

A female *Neoascia interrupta* (specimen ID Ox001311, ToLID idNeoInte1) was netted in Wytham Woods, Oxfordshire (biological vice-county Berkshire), UK (latitude 51.76, longitude –1.34) on 2021-04-23. The specimen was collected and identified by Steven Falk (University of Oxford) and preserved on dry ice.

The workflow for high molecular weight (HMW) DNA extraction at the Wellcome Sanger Institute (WSI) includes a sequence of core procedures: sample preparation; sample homogenisation; DNA extraction; HMW DNA fragmentation; and fragmented DNA clean-up. The sample was prepared for DNA extraction at the WSI Tree of Life laboratory: the dNeoInte1 sample was weighed and dissected on dry ice with tissue set aside for Hi-C sequencing ( https://dx.doi.org/10.17504/protocols.io.x54v9prmqg3e/v1). Tissue from the whole organism was disrupted using a Nippi Powermasher fitted with a BioMasher pestle ( https://dx.doi.org/10.17504/protocols.io.5qpvo3r19v4o/v1). DNA was extracted at the WSI Scientific Operations core using the Qiagen MagAttract HMW DNA kit, according to the manufacturer’s instructions.

All protocols developed by the Tree of Life laboratory are publicly available on protocols.io ( https://dx.doi.org/10.17504/protocols.io.8epv5xxy6g1b/v1).

### Sequencing

Pacific Biosciences HiFi circular consensus DNA sequencing libraries were constructed according to the manufacturers’ instructions. DNA sequencing was performed by the Scientific Operations core at the WSI on a Pacific Biosciences SEQUEL II (HiFi) instrument. Hi-C data were also generated from tissue of idNeoInte1 using the Arima2 kit and sequenced on the Illumina NovaSeq 6000 instrument.

### Genome assembly, curation and evaluation

Assembly was carried out with Hifiasm ( Cheng *et al.*, 2021) and haplotypic duplication was identified and removed with purge_dups ( Guan *et al.*, 2020). The assembly was then scaffolded with Hi-C data ( Rao *et al.*, 2014) using YaHS ( Zhou *et al.*, 2023). The assembly was checked for contamination and corrected as described previously ( Howe *et al.*, 2021). Manual curation was performed using HiGlass ( Kerpedjiev *et al.*, 2018) and Pretext ( Harry, 2022). The mitochondrial genome was assembled using MitoHiFi ( Uliano-Silva *et al.*, 2023), which runs MitoFinder ( Allio *et al.*, 2020) or MITOS ( Bernt *et al.*, 2013) and uses these annotations to select the final mitochondrial contig and to ensure the general quality of the sequence.

A Hi-C map for the final assembly was produced using bwa-mem2 ( Vasimuddin *et al.*, 2019) in the Cooler file format ( Abdennur & Mirny, 2020). To assess the assembly metrics, the *k*-mer completeness and QV consensus quality values were calculated in Merqury ( Rhie *et al.*, 2020). This work was done using Nextflow ( Di Tommaso *et al.*, 2017) DSL2 pipelines “sanger-tol/readmapping” ( Surana *et al.*, 2023a) and “sanger-tol/genomenote” ( Surana *et al.*, 2023b). The genome was analysed within the BlobToolKit environment ( Challis *et al.*, 2020) and BUSCO scores ( Manni *et al.*, 2021; Simão *et al.*, 2015) were calculated.

Table 3 contains a list of relevant software tool versions and sources.

**Table 3.** Software tools: versions and sources.

Software tool	Version	Source
BlobToolKit	4.1.7	https://github.com/blobtoolkit/blobtoolkit
BUSCO	5.3.2	https://gitlab.com/ezlab/busco
Hifiasm	0.16.1-r375	https://github.com/chhylp123/hifiasm
HiGlass	1.11.6	https://github.com/higlass/higlass
Merqury	MerquryFK	https://github.com/thegenemyers/MERQURY.FK
MitoHiFi	2	https://github.com/marcelauliano/MitoHiFi
PretextView	0.2	https://github.com/wtsi-hpag/PretextView
purge_dups	1.2.3	https://github.com/dfguan/purge_dups
sanger-tol/genomenote	v1.0	https://github.com/sanger-tol/genomenote
sanger-tol/readmapping	1.1.0	https://github.com/sanger-tol/readmapping/tree/1.1.0
YaHS	yahs-1.1.91eebc2	https://github.com/c-zhou/yahs

### Genome annotation

The BRAKER2 pipeline ( Brůna *et al.*, 2021) was used in the default protein mode to generate annotation for the *Neoascia interrupta* assembly (GCA_947623515.1) in Ensembl Rapid Release.

### Wellcome Sanger Institute – Legal and Governance

The materials that have contributed to this genome note have been supplied by a Darwin Tree of Life Partner. The submission of materials by a Darwin Tree of Life Partner is subject to the **‘Darwin Tree of Life Project Sampling Code of Practice’**, which can be found in full on the Darwin Tree of Life website here. By agreeing with and signing up to the Sampling Code of Practice, the Darwin Tree of Life Partner agrees they will meet the legal and ethical requirements and standards set out within this document in respect of all samples acquired for, and supplied to, the Darwin Tree of Life Project. 

Further, the Wellcome Sanger Institute employs a process whereby due diligence is carried out proportionate to the nature of the materials themselves, and the circumstances under which they have been/are to be collected and provided for use. The purpose of this is to address and mitigate any potential legal and/or ethical implications of receipt and use of the materials as part of the research project, and to ensure that in doing so we align with best practice wherever possible. The overarching areas of consideration are:

•   Ethical review of provenance and sourcing of the material

•   Legality of collection, transfer and use (national and international) 

Each transfer of samples is further undertaken according to a Research Collaboration Agreement or Material Transfer Agreement entered into by the Darwin Tree of Life Partner, Genome Research Limited (operating as the Wellcome Sanger Institute), and in some circumstances other Darwin Tree of Life collaborators.

## Data Availability

European Nucleotide Archive: *Neoascia interrupta* (bulrush Neoascia). Accession number PRJEB55984; https://identifiers.org/ena.embl/PRJEB55984 ( Wellcome Sanger Institute, 2022). The genome sequence is released openly for reuse. The *Neoascia interrupta* genome sequencing initiative is part of the Darwin Tree of Life (DToL) project. All raw sequence data and the assembly have been deposited in INSDC databases. Raw data and assembly accession identifiers are reported in Table 1.

## Author information

Members of the University of Oxford and Wytham Woods Genome Acquisition Lab are listed here: https://doi.org/10.5281/zenodo.7125292.

Members of the Darwin Tree of Life Barcoding collective are listed here: https://doi.org/10.5281/zenodo.4893703.

Members of the Wellcome Sanger Institute Tree of Life programme are listed here: https://doi.org/10.5281/zenodo.4783585.

Members of Wellcome Sanger Institute Scientific Operations: DNA Pipelines collective are listed here: https://doi.org/10.5281/zenodo.4790455.

Members of the Tree of Life Core Informatics collective are listed here: https://doi.org/10.5281/zenodo.5013541.

Members of the Darwin Tree of Life Consortium are listed here: https://doi.org/10.5281/zenodo.4783558.

## References

[ref-1] Abdennur N Mirny LA : Cooler: Scalable storage for Hi-C data and other genomically labeled arrays. *Bioinformatics.* 2020;36(1):311–316. 10.1093/bioinformatics/btz540 31290943 PMC8205516

[ref-2] Allio R Schomaker‐Bastos A Romiguier J : MitoFinder: Efficient automated large‐scale extraction of mitogenomic data in target enrichment phylogenomics. *Mol Ecol Resour.* 2020;20(4):892–905. 10.1111/1755-0998.13160 32243090 PMC7497042

[ref-3] Ball SG Morris RKA : Provisional atlas of British hoverflies (Diptera, Syrphidae).Huntingdon: Biological Records Centre, Centre for Ecology and Hydrology,2000. Reference Source

[ref-4] Ball S Morris R : Britain’s Hoverflies: A Field Guide –Revised and Updated Second Edition.Princeton: Princeton University Press,2015;17 Reference Source

[ref-5] Bernt M Donath A Jühling F : MITOS: Improved *de novo* metazoan mitochondrial genome annotation. *Mol Phylogenet Evol.* 2013;69(2):313–319. 10.1016/j.ympev.2012.08.023 22982435

[ref-6] Brůna T Hoff KJ Lomsadze A : BRAKER2: Automatic eukaryotic genome annotation with GeneMark-EP+ and AUGUSTUS supported by a protein database. *NAR Genom Bioinform.* 2021;3(1): lqaa108. 10.1093/nargab/lqaa108 33575650 PMC7787252

[ref-7] Challis R Richards E Rajan J : BlobToolKit - interactive quality assessment of genome assemblies. *G3 (Bethesda).* 2020;10(4):1361–1374. 10.1534/g3.119.400908 32071071 PMC7144090

[ref-8] Cheng H Concepcion GT Feng X : Haplotype-resolved *de novo* assembly using phased assembly graphs with hifiasm. *Nat Methods.* 2021;18(2):170–175. 10.1038/s41592-020-01056-5 33526886 PMC7961889

[ref-9] Di Tommaso P Chatzou M Floden EW : Nextflow enables reproducible computational workflows. *Nat Biotechnol.* 2017;35(4):316–319. 10.1038/nbt.3820 28398311

[ref-10] Guan D McCarthy SA Wood J : Identifying and removing haplotypic duplication in primary genome assemblies. *Bioinformatics.* 2020;36(9):2896–2898. 10.1093/bioinformatics/btaa025 31971576 PMC7203741

[ref-11] Harry E : PretextView (Paired REad TEXTure Viewer): A desktop application for viewing pretext contact maps. 2022; [Accessed 19 October 2022]. Reference Source

[ref-12] Howe K Chow W Collins J : Significantly improving the quality of genome assemblies through curation. *GigaScience.* Oxford University Press,2021;10(1): giaa153. 10.1093/gigascience/giaa153 33420778 PMC7794651

[ref-13] Kerpedjiev P Abdennur N Lekschas F : HiGlass: web-based visual exploration and analysis of genome interaction maps. *Genome Biol.* 2018;19(1): 125. 10.1186/s13059-018-1486-1 30143029 PMC6109259

[ref-14] Manni M Berkeley MR Seppey M : BUSCO update: Novel and streamlined workflows along with broader and deeper phylogenetic coverage for scoring of eukaryotic, prokaryotic, and viral genomes. *Mol Biol Evol.* 2021;38(10):4647–4654. 10.1093/molbev/msab199 34320186 PMC8476166

[ref-15] Rao SSP Huntley MH Durand NC : A 3D map of the human genome at kilobase resolution reveals principles of chromatin looping. *Cell.* 2014;159(7):1665–1680. 10.1016/j.cell.2014.11.021 25497547 PMC5635824

[ref-16] Rhie A McCarthy SA Fedrigo O : Towards complete and error-free genome assemblies of all vertebrate species. *Nature.* 2021;592(7856):737–746. 10.1038/s41586-021-03451-0 33911273 PMC8081667

[ref-17] Rhie A Walenz BP Koren S : Merqury: Reference-free quality, completeness, and phasing assessment for genome assemblies. *Genome Biol.* 2020;21(1): 245. 10.1186/s13059-020-02134-9 32928274 PMC7488777

[ref-18] Rotheray G : Colour Guide to Hoverfly Larvae (Diptera, Syrphidae) in Britain and Europe. *Dipterists Digest.* 1993;9:1–155. Reference Source

[ref-19] Simão FA Waterhouse RM Ioannidis P : BUSCO: assessing genome assembly and annotation completeness with single-copy orthologs. *Bioinformatics.* 2015;31(19):3210–3212. 10.1093/bioinformatics/btv351 26059717

[ref-20] Stubbs AE Falk SJ : British hoverflies: an illustrated identification guide.British Entomological and Natural History Society,2002. Reference Source

[ref-21] Surana P Muffato M Qi G : sanger-tol/readmapping: sanger-tol/readmapping v1.1.0 - Hebridean Black (1.1.0). *Zenodo.* 2023a; [Accessed 21 July 2023]. 10.5281/zenodo.7755665

[ref-22] Surana P Muffato M Sadasivan Baby C : sanger-tol/genomenote (v1.0.dev). *Zenodo.* 2023b; [Accessed 21 July 2023]. 10.5281/zenodo.6785935

[ref-23] Uliano-Silva M Ferreira JGRN Krasheninnikova K : MitoHiFi: a python pipeline for mitochondrial genome assembly from PacBio high fidelity reads. *BMC Bioinformatics.* 2023;24(1): 288. 10.1186/s12859-023-05385-y 37464285 PMC10354987

[ref-24] van Veen MP : Hoverflies of Northwest Europe: Identification keys to the Syrphidae.KNNV Publishing. Utrecht, Netherlands: BRILL,2014. Reference Source

[ref-25] Vasimuddin M Misra S Li H : Efficient Architecture-Aware Acceleration of BWA-MEM for Multicore Systems.In: *2019 IEEE International Parallel and Distributed Processing Symposium (IPDPS).*IEEE,2019;314–324. 10.1109/IPDPS.2019.00041

[ref-27] Wellcome Sanger Institute: The genome sequence of the bulrush Neoascia, *Neoascia interrupta* (Meigen, 1822). European Nucleotide Archive.[dataset], accession number PRJEB55984,2022.

[ref-26] Zhou C McCarthy SA Durbin R : YaHS: yet another Hi-C scaffolding tool. *Bioinformatics.* 2023;39(1): btac808. 10.1093/bioinformatics/btac808 36525368 PMC9848053

