# Supplementary material for: The genome sequence of the bulrush Neoascia, Neoascia interrupta (Meigen, 1822)
Source: Wellcome Open Res. 2023 Nov 23;8:549. [Version 1] doi: 10.12688/wellcomeopenres.20353.1 (PMC11101924; doi:10.12688/wellcomeopenres.20353.1)
Supplement: Peer review history

# Reviewer report: Reviewer response for version 1

**DOI:** 10.21956/wellcomeopenres.22538.r76316

**Published:** 2024-05-17

**Authors:** Iván Ballester-Torres

**Affiliations:** 1 Research Institute CIBIO (Centro Iberoamericano de la Biodiversidad), Science Park, University of Alicante, Alicante, Spain

The scientific note is clear and well-conducted. 

 I cannot make any comments about the methods of genome sequencing, as I have been working on barcoding and phylogenetic trees. 

 Regarding the keywords, I recommend to not repeat the same words written on the title, as well as not repeat the exact words on different keywords: " *Neoascia*".

 Regarding the Background section, it might be interesting to add a few words about the name of the plant species that adults of *N. interrupta* visit, and also the scientific names of the plants where larval development occur.

 The authors could mention the assessment of *N. interrupta *in the IUCN Red List of Hoverflies and its category (citation 1), in relation to the present study.

 Reference of the book [ref 1] the year of publication is incorrect. The book was published in 2004. The year of the reference must be changed in references section and also everytime is cited on the text.

Are sufficient details of methods and materials provided to allow replication by others?

Yes

Is the rationale for creating the dataset(s) clearly described?

Yes

Are the datasets clearly presented in a useable and accessible format?

Yes

Are the protocols appropriate and is the work technically sound?

Yes

Reviewer Expertise:

Entomology, Taxonomy and Systematics in Syrphidae, specifically in the genus Cheilosia

I confirm that I have read this submission and believe that I have an appropriate level of expertise to confirm that it is of an acceptable scientific standard.

## References

[1] 10.2305/IUCN.UK.2021-3.RLTS.T149171195A149171198.en

# Reviewer report: Reviewer response for version 1

**DOI:** 10.21956/wellcomeopenres.22538.r73549

**Published:** 2024-03-13

**Authors:** Luis Diambra

**Affiliations:** 1 Univeridad Nacional de La Plata, La Plata, Argentina

This manuscript from Falk and Woodcock reports the genome of an individual female of Neoascia interrupta. Raw genomic data was generated by Pacific Biosciences single-molecule HiFi. These long reads were assembled and scaffolded to Hi-C data. Haplotypic duplication was manually removed. The authors provide the assembly statistics obtained by using BlobToolKit Snailplo. The genome annotation was performed by BRAKER2 pipeline and includes 22,574 transcripts and 22,086 protein-coding genes. All these analyses are clearly explained in the protocols of Tree of Life laboratory which contains enough information to allow replication by other researchers.

 Main

 This Genome note provided a high-quality sequence dataset, and it contributes to the global resources for future discoveries by the scientific community.

 Minor

 I think that the authors could give more context about the importance (economic or social aspect) of this species, beyond that mentioned in the Introduction section.

Are sufficient details of methods and materials provided to allow replication by others?

Yes

Is the rationale for creating the dataset(s) clearly described?

Yes

Are the datasets clearly presented in a useable and accessible format?

Yes

Are the protocols appropriate and is the work technically sound?

Yes

Reviewer Expertise:

Bioinformatics

I confirm that I have read this submission and believe that I have an appropriate level of expertise to confirm that it is of an acceptable scientific standard.
